# Primary sarcomatoid urothelial carcinoma of the ureter: a case report and review of the literature

**DOI:** 10.1186/s12957-018-1383-9

**Published:** 2018-04-13

**Authors:** Yixiang Wang, Hanchao Liu, Ping Wang

**Affiliations:** Department of Urology, The Fourth Hospital Affiliated to China Medical University, Chongshan East street, Shenyang, Liaoning 110032 People’s Republic of China

**Keywords:** Sarcomatoid urothelial carcinoma, Ureter, Clinical treatment

## Abstract

**Background:**

Sarcomatoid urothelial carcinoma is a very dangerous malignant tumour derived from the epithelium. Primary sarcomatoid carcinoma of the ureter is extremely rare in clinical practice. The prognosis of this kind of disease is really poor, and there is still not a diagnosis standard in the world.

**Case presentation:**

An 82-year-old female patient who had intermittent waist pain without any other symptoms, had diagnosed as urothelial cancer on computerised tomography urography. Considering the patient’s age and quality of life, we made a preserving kidneys resection of the local tumour. The tumour was composed of sarcomatous and carcinomatous elements, and immunohistochemical examination showed that tumour cells were positive for cytokeratin, epithelial membrane antigen, vimentin, and GATA3 markers. There were no complications after 1-hour surgery. After 3 months, there was no signs of recurrence and metastasis.

**Conclusion:**

This case was a patient with sacomatoid urothelial carcinoma. Through a transurethral resection with laser, the patient recovered well, and there was no sign of any recurrence of the tumour after 3 months. With the development of technology and science, more and more cancerous patients’ living quality and survival rate were improved. Maybe it is essential for urologists and scientists to entirely understand the characteristics of the sarcomatoid urothelial carcinoma and make a better clinical guideline.

## Background

Sarcomatoid urothelial carcinoma is a very dangerous malignant tumour derived from the epithelium; its cells which are sarcomatoid differentiation can occur in any cancerous parts and more common in the respiratory system [1]. Primary sarcomatoid carcinoma of the ureter is extremely rare in clinical practice. This article reported a case of primary sarcomatoid urothelial carcinoma of the ureter and the clinical treatment. According to the best of our knowledge, sarcomatoid urothelial carcinoma occurring in the urinary system is very rare, especially in the ureter. In the urinary bladder, it only accounts for 0.1 to 0.3% of carcinomas [2]. And only 24 cases of the ureteral sarcomatoid urothelial carcinoma have been reported in the PubMed database [3]. Then, 11 adrenal sarcomatoid carcinoma cases have been reported from 1987 to 2016 [4]. The present case revealed a primary sarcomatoid urothelial carcinoma, pathology features and clinical treatments.

## Case presentation

**Fig. 1:**
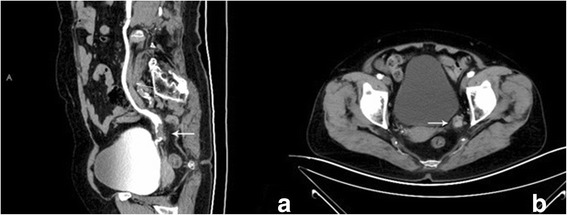
**a** Computed tomography urography shows an irregular mass in the left lower ureter. **b** The mass had a significant contrast enhancement during the nephrographic-phase

An 82-year-old female patient presenting with intermittent pain in the left flank was accepted by The Fourth Hospital Affiliated to China Medical University (LiaoNing, China) on April 19, 2017.On physical examination, the patient had no fever with a pulse rate of 88 beats/min and a blood pressure of 130/80 mmHg. There was mild left upper quadrant tenderness to palpation. She had no significant past medical or surgical history of note. She had a history of hypertension for 10 years. Laboratory examinations showed the following: white blood cell count, 4.70 × 109/l (normal range, 4.0–10.0 × 109/l); red blood cell count, 4.06 × 1012/l (normal range, 3.5–5.0 × 1012/l); haemoglobin level, 130 g/l (normal range, 110–150 g/l); red blood cells in urine, 1.6/HPF; and white blood cell in urine, 4.1/HPF. Cytological examination of the urine revealed no urothelial carcinoma, squamous cell carcinoma, or small cell carcinoma components. A computerised tomography (CT) urography demonstrated that a 9 × 7 mm irregular mass (31HU) was located in the lower ureter (Fig. 1a), which led to the dilatation of the proximal ureter and renal pelvis. During the nephrographic phase, the mass (82HU) had a significant contrast enhancement (Fig. 1b). Chest and abdomen computed tomography showed no evidence of distant visceral metastases. These led to suspicion of lower ureteral tumour.

**Fig. 2:**
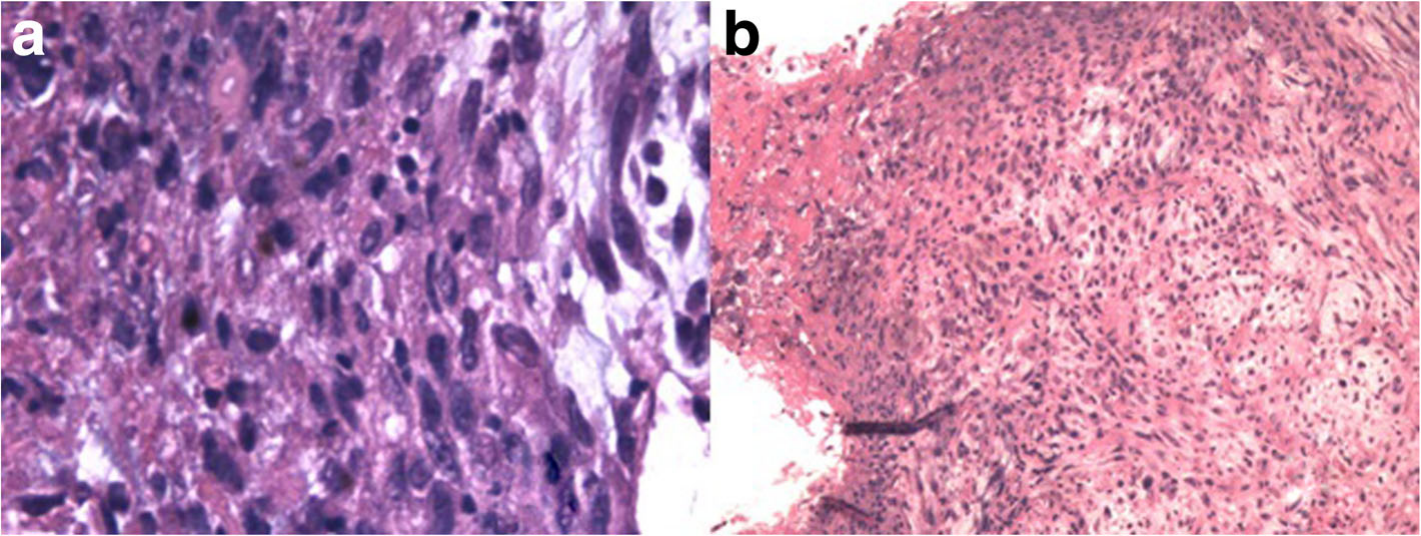
Histopathologic findings. The ureteral urothelial carcinoma merges with the sarcomatoid carcinoma component, disposed in large compact aggregates. HE, **a** × 100, **b** × 400

**Fig. 3:**
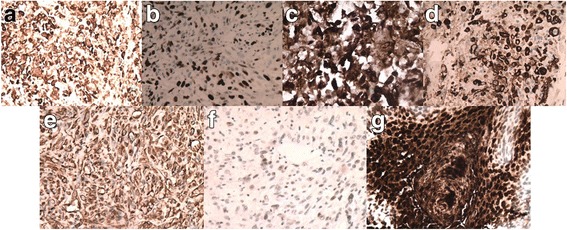
Immunohistochemical staining showing positivity for cytokeratin (**a**, × 200), carcinoembryonic antigen (**b**, × 200), vimentin (**c**, × 200), GATA (**d**, × 200), CKH (**e**, × 200), epithelial membrane antigen (**f**, × 400) in the tumour cells. A high proliferation index of the tumour components as shown by Ki-67 reactivity (**g**, × 200)

Trans-urethral resection of the tumour was carried out on April 27, 2017; due to the age and life quality of the patient, the ureter was retained. The tumour with a size of 1.0 cm in the lower part of the left lower ureter was removed by a 2 μm holmium laser and sent to the pathology department. Simultaneously, we used laser to cut the suspicious mucosa around the tumour and then indwelled double-J stent to prevent ureteral obstruction. Surgical samples were formalin-fixed, paraffin-embedded, and cut into 4 μm thick sections for the histological examination with hematoxylin-eosin stain and immunohistochemical procedures against epithelial membrane antigen (EMA), cytokeratin (CK), vimentin, desmin, carcinoembryonic antigen (CEA), cytokeratin high molecular weight (CKH), cytokeratins 20, S100, P63, GATA3, and Ki-67 (Table 1). The histopathological findings suggested that the tumour had a biphasic appearance with an epithelial component represented by areas of urothelial high grade carcinomas and a sarcomatoid component (Fig. 2). Immunohistochemical analysis indicated positivity of the tumour cells for vimentin, CK, EMA, CEA, CKH, GATA3, and P63 (Fig. 3a, b, c, d, e, f) and negativity for Desmin, S-100, and CK20. High proliferation index (40%) was documented against Ki-67 staining (Fig. 3g), indicating the malignant nature of the lesion. Based on the histological and immunohistochemical findings, sarcomatoid urothelial carcinoma was diagnosed. Due to patient and families rejected the adjuvant therapy, no postoperative radiotherapy or chemotherapy was performed. In July of this year, another ureterscope was carried to reexamine the situation where the tumour grew. It turned out to be good and there was no sign of any recurrence of the tumour.

**Table 1:** The results of the antibody panel

Antigen	Source	Clone	Pretreatment	CC	SRC
CK	KIT-0009	AE1/AE3	Trypsin	++	+
CEA	MAB-0043	ZC23	None	+	+
VIM	MAB-0735	MX034	PC-C	+	++
GATA 3	MAB-0695	L50-823	EDTA	+	+
CKH	KIT-0020	34βE12	PC-C	++	+
P63	MAB-0694	MX013	EDTA	+	–
EMA	KIT-0011	E29	None	++	+
Desmin	KIT-0023	D33	None	–	–
S-100	KIT-0007	4C4.9	PC-C	–	–
CK20	MAB-0669	MX003	PC-C	–	–
Ki-67	KIT-0005	MIB-1	PC-C	↑	↑

+: weak, ++: strong, −: negative, ↑: high proliferative activity. Trypsin: sections preheated with 0.1% trypsin (sigma) for 3 min at 37 °C. EDTA: sections preheated with ethylenediaminetetraacetic acid. PC-C: sections preheated in a conventional pressure cooker for 90 s in a citrate buffer solution

*CK* cytokeratin, *CEA* carcinoembryonic antigen, *VIM* vimentin, *CKH* cytokeratin high molecular weight, *EMA* epithelial membrane antigen, *CC* carcinomatous component, *SRC* sarcomatoid component

## Discussion

The urothelial carcinoma is the most common type of urinary cancers. However, sarcomatoid urothelial carcinoma is a highly malignant and rare form of urothelial carcinoma. And the histogenesis of sarcomatoid urothelial carcinoma is still a controversy issue. Two major hypotheses were presented. Some scientists consider that this type of tumour represents a collision tumour which is composed of two independent but simultaneously occurring epithelial and mesenchymal monoclonal neoplasms; others suggest that this carcinoma has a same clonal origin with branching differentiation into both components [5]. Most of the researchers consider that sarcomatoid carcinoma is one of the cancer metaplasia; its essence is still epithelial cancer cells which are sarcomatoid morphological differentiation. Epithelial components still express epithelial tissue markers cytokeratin, and sarcomatoid components express mesenchymal markers such as vimentin; two kinds of markers exist at the same time. The aetiology of sarcomatoid carcinoma is unclear. It may be derived from monoclonal tumour cells with epithelial and interstitial components which may be associated with 3,7,17 chromosome gene amplification and 9p21 chromosome deficiency [6]. Pathological characteristics are based on spindle cells with significant atypia, and some local tumour cells were epithelial differentiation. Most of them are high-grade urinary epithelial carcinoma. Immunohistochemistry is significantly helpful for the diagnosis, and the expression of epithelial and mesenchymal markers such as CK and vimentin is positive. And in this case, immunohistochemical features represent a typical sarcomatoid urothelial carcinoma features.

Ureteral sarcomatoid carcinoma often is found by physical examination, gross hematuria, or lower back pain. Because the illness was hidden, more than 85% of patients were found with the tumour for the T2 and T3 and accompanied by renal insufficiency. And the most of the frequent sites of metastasis were the regional lymph nodes, liver, lung and bone [7]. The present case described a woman patient who had a primary ureteral sarcomatoid carcinoma with no metastasis. The clinical symptom was just left flank pain. Imageology examination showed the hydronephrosis and occupied lesions. Compared with other cases, this one is maybe in an early stage of the tumour. The existing reliant literature is rare, and there are limited systemic options available to the best treatment option. According to the latest European Association of Urology Guidelines (2017 edition), radical nephroureterectomy remains the gold standard of the upper urinary tract urothelial carcinoma. RNU must comply with oncological principles, that is, preventing tumour seeding by avoidance of entry into the urinary tract during resection [8]. But the patient and her families refused this surgical option. In recent years, endoscopic ablation is also widely used, owing to the advantages of less trauma, less intraoperative blood loss, and protection of renal function. Compared to the upper urinary tract urothelial carcinoma, ureteral sarcomatoid urothelial carcinoma prognosis is poor and clinical guidelines is also insufficient. According to reports all over the world, ureteral sarcomatoid carcinoma lifetime is no more than 1 year [9]. According to the European urology clinical guideline, there are two major adjuvant treatment of urothelial carcinoma: (1) antegrade instillation of BCG vaccine or mitomycin C in the upper urinary tract by percutaneous nephrostomy and (2) retrograde instillation through a ureteric stent [8]. But the patient’s basic condition cannot take the risk of the two adjuvant treatments: bleeding, ureteric obstruction, and consecutive pyelovenous influx. At the same time, the patient and her families refused the adjuvant treatment. In this case, the patient was reexamed in July of 2017, and there was not any recurrence of the tumour. It may be due to the early diagnosis and therapy.

## Conclusion

In many kinds of the literature, some researchers believed the early diagnosis would contribute to a better prognosis. But according to the recently researches, there is still not a diagnosis standard in the world. Many researches found that the survival rate in kidney-sparing surgery is similar to radical nephroureterectomy in low-risk upper urinary tract urothelial carcinoma. But the sarcomatoid urothelial carcinoma is more malignant than urothelial carcinoma. It is necessary to study and pay attention to whether there is a significant difference in survival of sarcomatoid urothelial carcinoma cases. Maybe it is essential for urologists and scientists to entirely understand the characteristics of the sarcomatoid urothelial carcinoma and make a better clinical guideline. With the development of technology and science, more and more cancerous patients’ living quality and survival rate were improved.
